# SEOM clinical guidelines in early-stage breast cancer 2015

**DOI:** 10.1007/s12094-015-1427-3

**Published:** 2015-10-26

**Authors:** J. A. Garcia-Saenz, B. Bermejo, L. G. Estevez, A. G. Palomo, X. Gonzalez-Farre, M. Margeli, S. Pernas, S. Servitja, C. A. Rodriguez, E. Ciruelos

**Affiliations:** Hospital Clínico San Carlos, Madrid, Spain; Hospital Clínico Universitario, Valencia, Spain; CIOCC Hospital Universitario Madrid Sanchinarro, Madrid, Spain; Complejo Asistencial Universitario de León, León, Spain; Hospital Universitari Quirón Dexeus, Barcelona, Spain; Hospital Universitari Germans Trias I Pujol de Badalona (ICO-Badalona), Barcelona, Spain; Hospital Durán i Reynals (ICO), Barcelona, Spain; Hospital del Mar - Parc de Salut Mar, Barcelona, Spain; Hospital Universitario de Salamanca-IBSAL, Salamanca, Spain; Hospital Universitario 12 de Octubre, Madrid, Spain

**Keywords:** Early-stage breast cancer, Clinical guidelines, Adjuvant, Neoadjuvant, Genomic platforms

## Abstract

Breast cancer is a major public health problem. Despite remarkable advances in early diagnosis and treatment, one in three women may have metastases since diagnosis. Better understanding of prognostic and predictive factors allows us to select the most appropriate adjuvant therapy in each patient. In these guidelines, we summarize current evidence for the medical management of early-stage breast cancer.

## Introduction

Breast cancer is a major public health problem due to its high incidence, prevalence, and mortality. It is by far the most common cancer among women in Spain (2012), accounting for 29 % of all new cases of cancer in females. Moreover, it is the first cause of cancer-related mortality in the female population, accounting for 15.5 % of female cancer deaths, and the 5-year prevalence is 31.4 % [1].

Breast cancer is a heterogeneous disease with multiple intrinsic tumor subtypes [2]. Up to one in three patients will develop metastases during the disease depending on lymph node involvement and breast cancer subtype, despite remarkable progress in early diagnosis and treatment [3]. A better understanding of prognostic and predictive factors is allowing to individualize the treatment of early-stage breast cancer patients. The aim of these guidelines is to summarize current evidence and to give evidence-based recommendations for clinical practice.

## Methodology

These SEOM Guidelines have been developed with the consensus of ten breast cancer oncologists from the cooperative groups GEICAM (Spanish Breast Cancer Research Group) and SOLTI (Spanish Collaborative Group for the Study, Treatment and Other Experimental Strategies in Solid Tumors). To assign a level of evidence and a grade of recommendation to the different statements of this treatment guideline, it was decided to use the Infectious Diseases Society of America-US Public Health Service Grading System for Ranking Recommendations in Clinical Guidelines to determine the quality of evidence and strength of recommendation in each of the consensus recommendations (Table 1).

**Table 1:** Strength of recommendation and quality of evidence score

Category (grade)	Definition
Strength of recommendation	
A	Good evidence to support a recommendation for use
B	Moderate evidence to support a recommendation for use
C	Poor evidence to support a recommendation
D	Moderate evidence to support a recommendation against use
E	Good evidence to support a recommendation against use
Quality of evidence	
I	Evidence from ≥1 properly randomized, controlled trial
II	Evidence from ≥1 well-designed clinical trial, without randomization; from cohort or case-controlled analytic studies (preferably from >1 center); from multiple time series; or from dramatic results from uncontrolled experiments
III	Evidence from opinions of respected authorities, based on clinical experience, descriptive studies, or reports of expert committees

## Diagnosis and initial workup

The following tests allow for a correct diagnostic and prognostic approach to all patients in whom the presence of a breast tumor is suspected.

- Mammography and ultrasound: Initial imaging test which also permits a biopsy to be taken of suspicious lesions [I, A].
- Core biopsy: essential for diagnosis and to obtain prognostic information. It is essential to study the estrogen receptor, progesterone receptor, and HER2. Ki-67 determination has high inter-observer variability and care should be taken when using Ki67 to inform the decision-making process [I, A] [4].
- MRI: allows for better staging of the disease by detecting disease foci not visible by other methods. Additional findings must be confirmed histologically due to the rate of false positives. Use of MRIs has not shown a survival benefit, and therefore, it is not considered a mandatory test [I, B] [5]. It should be used in case of nodal carcinoma or Paget’s disease without lesion identifiable in mammogram or ultrasound. It can be useful prior and after neoadjuvant treatment to define the extent of disease and monitor the response to treatment.
- Additional studies: anamnesis, complete physical examination, lab test with complete blood count, liver and renal function, alkaline phosphatase and calcium. When anomalies are detected in these tests, or when disease is detected at advanced stage (stage III), a more extensive study is performed using PET-CT or thoracic-abdominal CT and bone scan (if bone symptoms, elevation of alkaline phosphatase, LDH, or calcium are present) [I, B] [6].

## Principles of surgery in early-stage breast cancer

Breast-conserving surgery based on lumpectomy is equivalent to mastectomy and must be considered as first option in most cases of stages I–II [Level of evidence, Grade of recommendation A] [7, 8]. Lumpectomy is not indicated in case of diffuse disease in the breast, and in cases with positive margins when re-excision shows residual disease or it can not be performed with adequate cosmetic results.

Additionally, other relative contraindications for lumpectomy are previous radiation therapy in the breast, active connective diseases with skin involvement, or tumors with diameter greater than five cm when neoadjuvant treatment is not indicated or has been not effective. Radiation therapy during pregnancy is contraindicated, so mastectomy must be considered if locoregional treatment must be completed before childbirth.

When mastectomy is performed, contralateral mastectomy as a prophylactic procedure is not indicated in most of the patients. Younger women, in which BRCA mutations are more common, can benefit most from this strategy. However, decision of contralateral mastectomy must be considered only after an adequate process of counseling and discussion of risk–benefit [9].

Sentinel lymph node (SLN) mapping is recommended for assessment of the involvement of axillary lymph nodes and should be performed in patients with clinically negative axillary nodes (Level of Evidence I, Grade of recommendation A). In patients with clinically positive axillary nodes, pathologic confirmation must be done by fine needle aspiration (FNA) or core biopsy to confirm pathologic involvement.

Axillary lymph node dissection (ALND) is indicated in patients with positive SLN biopsy or confirmed preoperative pathologic axillary lymph node involvement. However, in patients with stage I-II disease and less than three positive axillary nodes after SLN biopsy and lumpectomy (and adjuvant radiotherapy indicated), ALND can be avoided without significant negative impact in DFS and OS when an adequate postoperative treatment is provided [I, A] [10].

Regarding SLN biopsy in patients receiving treatment with neoadjuvant (primary systemic) therapy, ALND is recommended for cases with pathologically confirmed axillary lymph node involvement by FNA or core biopsy before neoadjuvant treatment. In patients with clinically positive axillary nodes before neoadjuvant and negative SLN biopsy after chemotherapy, the rate of false negative is higher than 10 % and ALND is recommended as standard procedure. However, some studies indicated that for these patients, when at least three sentinel nodes are identified and all of them are negative, ALND can be avoided (Level of evidence II, Grade of recommendation B). For cases with clinically negative axillary nodes, SLN biopsy can be performed before or after neoadjuvant treatment [11].

## Principles of adjuvant systemic therapy. Genomic profiles in decision-making in systemic adjuvant treatment

Systemic adjuvant treatment is commonly used in early breast cancer with the intention to reduce the rate of locoregional or systemic relapses a death derived from the disease. Unfortunately, treatment decisions are based on statistical models in which patients have indirect estimations of the risk of relapse and death, and therefore of the potential benefits of implementing adjuvant strategies.

Multigenic tests provide information beyond standard clinical and pathologic prognostic factors that can help in making treatment decisions in ER-positive breast cancer. All of these tests have been validated for prognosis in year 1–5, but there are some differences, especially beyond 5 years and regarding predicting usefulness of chemotherapy. We reviewed validation studies of the most commonly used tests in our country with level of evidence according to Simon et al. [12].

Oncotype DX. It has been validated for predicting risk of distance recurrence to 10 years in breast cancer patients treated with hormone therapy. Oncotype has also proved predictive value of the benefit of chemotherapy in two randomized studies (NSABP B-20 and SWOG) [13–15]. In the German prospective study Plan B, high-risk patients according to classical clinicopathological factors, in case of positive ER, and 0–3 nodes and RS of 11 or less, were treated with hormone therapy alone; results recently presented showed a 3 years event-free survival of 98.3 %, in spite of features of high risk by the traditional parameters [16]. In a recently report, based on studies NSABP B-28 and B-14, RS was associated with 5–15 years distant relapse risk in 86 % of patients, who had expression of RE greater than 0.9 [17]. Recently, a prospectively conducted study concluded that ER+, HER2- N0 patients with tumors that had a favorable gene expression profile had very low rates of recurrence at 5 years with endocrine therapy alone [18]. Level of evidence: IA for the prediction of adjuvant chemotherapy benefit in low risk group patients. IB for the prediction of the risk of distant recurrence at 10 years and benefit of adjuvant chemotherapy in the other ER-positive, HER2-negative groups.

PAM50 ROR. The PAM50 gene signature classify a tumor as one of four intrinsic subtypes (Luminal A, Luminal B, HER2-enriched, and Basal-like), which have been shown to be prognostic in both untreated and tamoxifen-treated patient populations. PAM50 estimates patient’s probability of disease recurrence by weighting the molecular subtype correlations, a subset of proliferation genes, and pathologic tumor size [19, 20]. PAM50 ROR score has clearly demonstrated its prognostic value beyond 5 years and added significant prognostic information beyond the Oncotype RS in ER-positive, postmenopausal patients treated with endocrine therapy alone (from the ATAC trial) [21]. It has demonstrated additional prognostic value in node-positive patients for the risk of late recurrence on patients from the ABCSG-8 trial [22, 23]. In a recent report, ROR obtained from core needle biopsy predicted response to neoadjuvant chemotherapy in hormone-sensitive HER2-negative patients [24]. Level of evidence: IB for the prediction of the risk of recurrence 10 years away.

Mammaprint: this signature was validated in 295 patients with pT1 or pT2 tumors with pN0 or pN+, with a median follow-up of 7.8 years, showing a prognostic value. This prognostic value has been validated in other studies, including patients with up to 4–9 positive lymph nodes [25, 26]. Level of evidence: IB for prognostic value.

EndoPredict. EPclin (EndoPredict combined with nodal status and tumor size) was validated in two randomized phase III trials, showing prognostic value, for year 1-5 and beyond [27, 28]. Level of evidence: IB for prognostic value.

For patients with ER+/HER2- tumors, multigenic tests appear to identify a group of very good prognosis patients for whom, the benefits of chemotherapy are so small that they do not outweigh the risks. The assays reviewed have their own individual advantages. Oncotype, Mammaprint, PAM-50 ROR score, and EndoPredict are useful in predicting relapse risk for year 1–5. Beyond 5 years Oncotype, EndoPredict has showed prognostic value. But PAM-50 ROR showed the strongest prognostic value beyond 5 years. Oncotype showed the strongest predictive value of the benefit of chemotherapy [29].

## Systemic treatment for Luminal-type early-stage breast cancer

### Adjuvant endocrine therapy for early-stage breast cancer

There is robust evidence that endocrine therapy improves survival of early-stage luminal breast cancer. Therefore, adjuvant hormonotherapy should be offered to any of these patients regardless of age, comorbidity, risk, menopausal status, chemotherapy exposure, expression level of ER, PgR expression, and/or Her2 status [I, A].

There are several endocrine treatment options. The choice would be adjusted to menopause status, comorbidity, and the risk of recurrence. There is not a universal consensus to determine the risk of recurrence. It is assumed that those patients whose tumors exhibit a high-risk pathological and/or genotypic profile established by genomic platforms to advise the use of adjuvant chemotherapy should be considered at high risk of recurrence [III, D].

The standard treatment for premenopausal women is 5 years of tamoxifen (I, A) [30], but other alternatives should be considered. Extending tamoxifen beyond 5 years confers a significant reduction in risk of recurrence but questionable specific mortality at 10 years, according to the ATLAS and ATTOm studies [31, 32]. Both studies concluded that the benefit is possibly higher in high-risk tumors but at the cost of greater toxicity [I, B]. Adjuvant exemestane with ovarian suppression as compared with tamoxifen plus ovarian suppression significantly reduced recurrence but did not improve overall survival in high-risk adjuvant-chemotherapy-treated premenopausal breast cancer patients [33]. This combination is an alternative to tamoxifen for patients at high risk of recurrence, at the cost of greater toxicity [I, B].

EBCTCG and SOFT trials support that adjuvant tamoxifen with GnRH analogues is not better but more toxic than tamoxifen alone. This combination could be seen as an alternative for high-risk patients who cannot tolerate the use of aromatase inhibitors [II, B].

The standard treatment for postmenopausal women is an aromatase inhibitor sometimes during adjuvant treatment. There is not a better than another inhibitor (I, A). Tamoxifen is the second choice (II, B). Front line aromatase inhibitor therapy should last for 5 years. In tamoxifen-starting patients, the switch to aromatase inhibitor can be offered after 2–3 years. Aromatase inhibitor can also be offered beyond 5 years of tamoxifen.

### Primary/neoadjuvant therapy for hormone receptor-positive disease

In locally advanced cancer and large tumors, primary systemic treatment is indicated (I, A). Systemic treatment (chemotherapy or endocrine therapy) allows to reduce the extent of surgery. All treatments recommended in the adjuvant setting (chemotherapy, endocrine therapy, and targeted therapies) may also be used in the preoperative setting.

If chemotherapy is used, it is recommended to deliver all the chemotherapy before surgery, without breaks, with the aim to increase the probability of achieving a pCR, a proven factor for good prognosis. A sequential regimen of anthracyclines and taxanes (6–8 cycles) was associated to increased probability of pCR, and must be recommended [II, B] [34, 35].

For ER-positive and Her2-negative disease, especially for lobular subtype or luminal A tumors (less responsive to chemotherapy), endocrine therapy given for 6 months is a good option [36]. For postmenopausal women, AIs are more effective than tamoxifen [I, A] [37, 38]. There are no trials evaluating the role of neoadjuvant hormonal treatment in premenopausal patients.

## Systemic therapy for early-stage triple-negative breast cancer

### Adjuvant treatment for triple-negative disease

Triple-negative breast cancer (TNBC) is a heterogeneous disease comprising approximately 15 % of all breast cancers. With the exception of medullary, adenoid cystic, and apocrine carcinomas that have a better outcome, TNBCs have generally an aggressive behavior. Molecular profiling has demonstrated that TNBC encloses 6 distinct biological subtypes that reflect a wide heterogeneity in these tumors.

Conventional chemotherapy remains the mainstay of adjuvant systemic treatment for most patients with early TNBC. Adjuvant chemotherapy should include an anthracycline and a taxane [I, B]. Nevertheless, women with T1a/bN0 tumors have an excellent prognosis without chemotherapy [39]. No adjuvant chemotherapy is recommended in tumors <0.5 cm (pT1a), and for 0.6–1 cm tumors, it has to be discussed and balanced [III, B]. The incidence of BRCA mutations is higher among TNBC patients (approximately 20 %), and 90 % of BRCA1-mutated tumors are TNBC, but there is not a different treatment option for mutated patients, at present [III, C].

### Neoadjuvant treatment for triple-negative disease

Neoadjuvant therapy in TNBC leads to pathologic complete response (pCR) rates of 30–40 %, which has been associated with an excellent prognosis [40]. However, the majority of TNBC patients do not achieve a pCR and have a high risk of early relapse and poor prognosis. The use of platinum-based chemotherapy in TNBC is currently being evaluated. Neoadjuvant cisplatin monotherapy in BRCA1 mutation-breast cancer patients is highly effective with pCR rates of 72–83 %. BRCAness and basal-like subtype may also predict response to DNA-damaging agents.

Two randomized phase II neoadjuvant trials [41, 42] and a meta-analysis [43] have assessed the addition of carboplatin to anthracyclines and taxane-based chemotherapy for TNBC. In the GeparSixto trial, the addition of carboplatin significantly increased the pCR rate in TNBC patients treated with weekly paclitaxel and liposomal doxorubicin plus bevacizumab. Patients with both a BRCA1/2 and family history of breast or ovarian cancer had the largest improvement in pCR. In the CALGB 40603 study, the addition of carboplatin (at a dose of AUC every 3 weeks), to weekly paclitaxel followed by AC every 2 weeks, increased the rate of pCR as well. Nevertheless, the addition of platinum results in added toxicity, and it remains unclear how to be use them and whether platinum-based chemotherapy can extend the survival rate of TNBC patients.

Regarding the type of taxane and outcome, an improved pCR was observed with Nab-paclitaxel compared to solvent-based weekly paclitaxel (43 vs 34 %) in a head to head phase III neoadjuvant trial. This effect was seen in all subgroups specially in TNBC patients [44].

Recommendation: Sequential regimens of anthracyclines followed by taxanes are the standard treatment. The use of platinum for the neoadjuvant treatment of TNBC is not currently recommended except for BRCA mutation carriers or patients with a strong family history of breast cancer [I, A].

## Systemic treatment for early-stage HER2-positive breast cancer

### Adjuvant treatment for HER2-positive disease

HER2 receptor is a membrane tyrosine kinase and when activated affects cell proliferation and survival. HER2 oncogene amplification is the primary pathway of HER2 receptor overexpression and is a major driver of tumor development and progression in a subset of breast cancers. The overexpressed HER2 receptor is a valuable therapeutic target.

Trastuzumab is a humanized monoclonal antibody with the specificity for the extracellular domain of HER2. The scientific evidence is definitive to recommend adjuvant trastuzumab in node-positive and node-negative tumors sized >1 cm [I, A] [45, 46]. Due to the relatively high failure risk, even in patients with node-negative tumors measuring 0.6 to 1.0 cm, it should also be considered, particularly in ER-negative disease (II, B) [47]. In most studies, trastuzumab was administered for 1 year. No additional benefit was demonstrated for 2 year further administration [I, A] [48].

Due to its cardiotoxicity, trastuzumab should not be routinely administered concomitantly with anthracyclines (I, B). Combination with taxanes is safe and has been demonstrated to be more effective than sequential treatment. Both AC followed by paclitaxel/docetaxel with trastuzumab for 1 year commencing with the first dose of taxanes or TCH are preferred regimens [49].

Luminal B HER2-positive tumors are treated with chemotherapy, endocrine therapy, and trastuzumab. No randomized data exist to support omission of chemotherapy in this group. However, in small, node-negative tumors, combination of single-agent paclitaxel and trastuzumab provides excellent results [II B] [50]. Trastuzumab may also be safely combined with either radiotherapy or endocrine therapy.

### Neoadjuvant treatment for Her2-positive disease

In the neoadjuvant setting, trastuzumab in combination with chemotherapy (taxane and anthracycline based) has been the standard treatment for HER2-positive tumors with axillary involvement or sized ≥2 cm [51]. Dual anti-HER2 blockade has lead to higher rates of pathological complete responses but with no improvements in long-term outcomes. Dual antiHER2 blockade associated with chemotherapy (trastuzumab + lapatinib, trastuzumab +pertuzumab) has led to improvements in the pCR rate when compared with chemotherapy associated with trastuzumab agent, as shown in NEOSPHERE study. These data have been key to the accelerated approval of this combination, still dependent on the results of an adjuvant confirmatory trial [II B] [52]. Lapatinib–trastuzumab combo did not translate into improvement in long-term outcomes, either in the adjuvant or neoadjuvant setting and such a treatment cannot be recommended [I, A] [53, 54].

## Recommendations of adjuvant radiotherapy

Adjuvant radiotherapy (RT) should be performed in the case of:

- Breast-conserving surgery: external beam whole radiation therapy (WBRT). If four or more nodes are involved, supra and infraclavicular radiotherapy is recommended [I, A].
- Mastectomy: chest wall radiation and supra and infraclavicular nodes with or without internal mammary and axillary nodes in patients with T4, T3 N+ and involvement of four or more nodes [I, A].
- In one to three involved nodes after breast-conserving surgery, supra and infraclavicular nodal irradiation is recommended to minimize the risk of recurrence and potentially improve disease-specific survival [I, B] [55].
- Patients with T1-2 and one to three involved nodes and T3N0 after mastectomy have an increased risk of a locoregional recurrence. However, the benefit of adjuvant RT can be small, and must be discussed with the radiation oncologist and other risk factors such a high grade, age, or lymphovascular invasion must be taken into account [I, B] [56].

Radiotherapy treatment is generally well tolerated, with minimal toxicity in the medium and long term, with planning and management methods of treatment introduced in recent years. In the indications shown, radiation therapy has proved effective and very relevant in decreasing the risk of local recurrence and improving breast cancer mortality. Despite these recommendations, it is important to note that in elderly patients with stage I, specific studies have not shown improved survival and should therefore be assessed individually [I, A] [57]. Furthermore, some low-risk patients treated with conservative surgery could be spared whole breast RT and receive only partial breast radiation or intraoperative treatment, although less evidence exists to support this approach
